# Psychosomatic Disorders, Epigenome, and Gut Microbiota

**DOI:** 10.3390/cells14241959

**Published:** 2025-12-10

**Authors:** Hamid Mostafavi Abdolmaleky, Ahmad Pirani, Giuseppe Pettinato

**Affiliations:** 1 Department of Medicine (Biomedical Genetics), Chobanian & Avedisian School of Medicine, Boston University, Boston, MA 02118, USA; 2 Department of Medicine, Beth Israel Deaconess Medical Center, Harvard Medical School, Boston, MA 02215, USA; gpettina@bidmc.harvard.edu; 3 Mental Health Research Center, Psychosocial Health Research Institute, Iran University of Medical Sciences, Tehran 14535, Iran

**Keywords:** psychosomatic disorders, mental distress, gut microbiota, epigenetics

## Abstract

**Highlights:**

**What are the main findings?**

- Patients with psychosomatic disorders exhibited mental distress, gut dysbiosis, and aberrant gut microbiota (GM) profiles that contribute to the severity of disease via epigenetic mechanisms.
- Probiotic supplements and other gut-balancing therapies could serve as promising approaches for treating psychosomatic disorders by mitigating epigenetic aberrations.

**What are the implications of the main findings?**

- The intercommunication system between the gut and the brain, known as the gut–brain–microbiota axis, plays a crucial role in pathogenesis of psychosomatic disorders
- GM based interventions such as “prebiotics, probiotics, synbiotics, postbiotics, and fecal microbiota transplantation” may contribute to improving physical and psychological symptoms in patients with psychosomatic disorders by replenishing the abnormal GM composition and enhancing concentrations of beneficial epigenetic metabolites.
- Human iPSC-derived multicellular organoids may serve as powerful platforms for therapeutic interventions using probiotic supplements and for unraveling mechanistic pathways underlying inter-organ interactions.

**Abstract:**

Psychosomatic disorders are conditions in which physical (somatic) symptoms are triggered or aggravated by psychological distress. These disorders result from complex interactions among the endocrine, central nervous, and immune systems. Emerging evidence indicates that gut microbiota (GM) dysbiosis, epigenetic alterations, and immune system dysregulation play pivotal roles in the pathogenesis of psychosomatic disorders and may serve as potential biomarkers for disease states and therapeutic outcomes. This review first outlines how epigenetic dysregulation contributes to psychosomatic disorders through altered expression of genes such as GRM2, TRPA1, SLC6A4, NR3C1, leptin, BDNF, NAT15, HDAC4, PRKCA, RTN1, PRKG1, and HDAC7. We then examine current evidence linking psychosomatic disorders with changes in GM composition and GM-derived epigenetic metabolites, which influence immune function and neurobiological pathways. The core focus of this review is on therapeutic interventions—including probiotics, prebiotics, postbiotics, fecal microbiota transplantation, and targeted dietary approaches—that modulate the gut–brain axis through epigenetic mechanisms for the management of psychosomatic disorders. Finally, we highlight the current challenges and future directions in elucidating the interplay between epigenetics, the GM, and psychosomatic disease mechanisms. In this context, human iPSC-derived multicellular organoids may serve as powerful platforms to unravel mechanistic pathways underlying inter-organ interactions.

## 1. Introduction

Psychosomatic disorders are diseases with physical manifestations caused or aggravated by psychological factors such as stress, anxiety, depression, trauma, and emotional distress [1]. Psychosomatic disorders involve a link between mind and body in which changes in mental states influence physical health and performance and impair quality of life [2,3]. These disorders are defined as conditions in which interactions between psychological, neuroendocrine, and immune systems affect the onset, severity, or longevity of physical symptoms and are marked not by symptoms that are “psychological” or “imagined” but by bidirectional mind–body mechanisms that lead to considerable alterations in physiological function [3,4]. Despite their clinical heterogeneity, psychosomatic disorders like irritable bowel syndrome (IBS), fibromyalgia, chronic fatigue syndrome, functional neurological diseases, and gastrointestinal (GI) disturbances resulting from stress can be categorized under this umbrella owing to overlapping mechanisms, including disruptions in the hypothalamic–pituitary–adrenal (HPA) axis [5,6], autonomic balance [7], and immune function [8,9] accompanied with abnormal changes in the gut microbiota (GM) composition [10].

Stressful situations like noise, pollution, and childhood trauma are responsible for the etiopathogenesis of psychosomatic disorders [11,12]. Physical symptoms may be related to various body organs, including heart (elevated heart rate and hypertension), lung (shortness of breath), GI tract (nausea and vomiting), muscle (pain), and skin [4]. Stress is associated with physiological adaptive responses to manage the stressors that disrupt internal balance. Chronic exposure to stressors may lead to sustained activation of the central sympathetic nervous system and the HPA axis, which, in turn, escalate release of cortisol and subsequently give rise to adverse health impacts like obesity, mental disorders, and cardiovascular diseases [13,14,15]. Psychological risk factors, such as depression and negative personality traits, can also contribute to the exacerbation of injury or even death in patients with cardiovascular diseases (CVDs) [16,17]. Subjects with fibromyalgia and rheumatoid arthritis show a high prevalence of comorbid psychiatric symptoms, both at clinical and subclinical levels, which complicates disease pathogenesis, indicating the need for considering biological, psychological, and social interventions in the treatment process [18,19,20].

Stress plays a key role in influencing eating behavior, as it can activate reward center pathways in the brain, leading to overeating unhealthy foods [13]. Reciprocally, subjects with digestive disorders are more susceptible to symptoms of anxiety, depression, and other mental disorders. For example, Feng et al. reported that patients with GI diseases exhibited 20.74% anxiety symptoms alone, 31.78% depressive-like behaviors alone, 13.99% both anxiety and depressive-like behaviors, and 38.53% either depressive-like behaviors or anxiety symptoms [21]. Additionally, more than 50% of subjects with IBS exhibit depressive and anxiety symptoms, panic attacks, sleep problems, and loss of appetite [22,23]. Zamani et al. reported that subjects with IBS also exhibit three-fold elevated odds of either anxiety or depression versus healthy controls [24].

As multiple lines of evidence link psychosomatic disorders to alterations in the GM, it is important to recognize that the human microbiota represents a highly dynamic and complex micro-ecosystem, comprising trillions of host-adapted microorganisms, including bacteria, viruses, fungi, and diverse microbial and eukaryotic species. Within this community, bacteria are the predominant inhabitants, comprising approximately 99% of the total microbial population in the gastrointestinal tract. Large-scale initiatives such as MetaHit and the Human Microbiome Project have identified more than 2000 microbial species residing in the human gut [25]. The normal GM is mainly dominated by four major bacterial phyla, including *Firmicutes*, *Bacteroidetes*, *Proteobacteria*, and *Actinobacteria*, which totally compose 98% of its population [26]. This microbial consortium is a key player in numerous physiological processes, including maturation of the immune system, regulation of immune homeostasis, contributing to well-coordinated immune responses, protection against pathogens, maintenance of the intestinal barrier integrity, and regulation of metabolism and energy balance [27,28]. Maintaining a healthy and resilient GM is critical, as it supports the microbial community’s ability to restore homeostasis following disruptions caused by poor dietary habits, excessive use of antibiotics and other medications, infections, and various environmental factors [29,30,31]. When the GM fails to resist or recover from such insults, abnormal shifts in its composition may occur, leading to an imbalanced state known as gut dysbiosis [32,33]. This condition is characterized by alterations in microbial composition, including increased abundance of pro-inflammatory taxa and reduced abundance of anti-inflammatory or commensal species [34,35]. Persistent dysbiosis is capable of destabilizing multiple physiological regulatory systems, eventually driving both localized and systemic inflammatory responses [36,37]. Gut dysbiosis can also compromise intestinal barrier integrity by increasing intestinal permeability, commonly referred to as “leaky gut” [38,39,40]. Leaky gut facilitates translocation of microbial components like lipopolysaccharide (LPS) into the blood circulation, which, in turn, results in initiation of immune activation and the secretion of inflammatory mediators, including IL-6 and TNF-α [41,42,43]. These cytokines can penetrate the blood–brain barrier (BBB) or signal through neural pathways, where they are capable of regulating the activity of epigenetic enzymes such as DNA methyltransferases and histone deacetylases, as well as modulating microRNA expression [44,45]. These abnormalities can affect neural gene expression and function, thereby linking gut dysbiosis and increased intestinal permeability to alterations in brain plasticity, mood regulation, and behavior [46,47].

In the context of the relationship between GI diseases and psychiatric conditions, it is important to note that over 90% of the body’s serotonin, a neurotransmitter involved in mental health, is produced in the digestive tract by enterochromaffin cells of the intestinal mucosa and certain bacteria, such as *Lactobacillus* species and *Lactiplantibacillus plantarum*, which influence its production [48,49]. Therefore, gut dysbiosis may contribute to developing mental disorders by changing the GM composition and disrupting production, secretion, and reuptake of neurotransmitters [50]. Likewise, aberrations in the composition of GM-derived epigenetic metabolites due to gut dysbiosis and environmental factors may accelerate the progress of psychiatric conditions like depressive-like behaviors [51]. As an example, depletion of ghrelin (a hormone produced in the stomach that plays a crucial role in stimulating appetite and regulating energy balance) elevates intestinal permeability, depressive-like behaviors, and inflammation in experimental ulcerative colitis in aged mice by reducing tryptophan and its microbial derivatives indole-3-acetic acid and indole-3-lactic acid [52]. Moreover, stress is one of the factors which affect both the development of psychosomatic disorders like IBS and intensify the severity of the GI symptoms in these disorders [22]. As an example, Scaldaferri et al., in a five-year follow-up study, found an interesting link between psychopathological traits, such as obsessiveness, health concerns, somatization, and reduced abundance of short-chain fatty acid (SCFA)-producing bacteria at baseline that can serve as predictive factors to evaluate ulcerative-colitis-related adverse outcomes [53].

The scope of this review will highlight the roles of GM microbial composition and GM-derived epigenetic metabolites involved in epigenetic dysregulations linked to the etiopathogenesis of psychosomatic disorders. Among the core focuses of this review are therapeutic strategies, including probiotics, prebiotics, postbiotics, fecal microbiota transplantation, and certain diets that may modulate the gut–brain axis via epigenetic mechanisms for the management or treatment of psychosomatic disorders. The prospects and challenges in investigating the critical role of epigenetics and the GM in psychosomatic disorders are proposed in the final section.

## 2. Gut Microbiota Dysbiosis and Neuropsychiatric Symptoms in Patients with Psychosomatic Disorders

As noted earlier, the human GM is a complex community of trillions of microorganisms residing in the GI tract, serving as key contributors to digestion, metabolism, immune, and central nerve system (CNS) functions [54,55]. The GI tract via endocrine, immune, metabolic, and neural pathways dynamically communicates with the CNS, collectively forming the gut–brain axis [56,57]. This communication is mediated by the vagus nerve and the enteric nervous system. The vagus nerve establishes a connection between the brainstem and the gut and contributes to the transition of signals that affect mood, cognition, and stress responses [58]. The enteric nervous system also contributes to regulation of GI functions and establishes connections between the gut and CNS via sensory and motor pathways [59].

A growing body of evidence has indicated that patients with psychosomatic disorders exhibited remarkable changes in GM profiles, characterized by decreased microbial diversity and an overgrowth of pathogens and pro-inflammatory species [60]. In regard to a causal relationship between gut dysbiosis and disease development, transfer of Crohn’s colitis microbiota to germ-free mice has been shown to induce chronic Crohn’s-like colitis based on histology, inflammatory cytokine expression, and transcriptomic changes [61]. Furthermore, fecal microbiota transplantation from patients with IBD and comorbid depression could modify immune responses and induce depression-like behavioral changes in recipient mice [62]. Abnormal changes in the GM composition also correlate with the severity of mental and physical symptoms in patients with psychosomatic disorders [63]. The co-occurrence of diseases like IBS with neuropsychiatric disorders indicates that the gut–brain axis is one of the crucial factors in the pathogenesis of these diseases [64]. On the other hand, psychological factors like stress are capable of decreasing microbial diversity in the gut, reducing abundance of beneficial bacteria, such as *Lactobacillus* and *Bifidobacterium*, which are involved in maintaining gut barrier integrity and immune modulation by producing epigenetic metabolites such as butyrate and acetate [65,66]. This depletion of commensal bacteria reduces the gut’s defense and contributes to increasing growth and activity of harmful species, like the *Proteobacteria* phylum, that are involved in inflammation and disease [66,67,68]. Therefore, psychological stress not only alters the composition of the GM and increases intestinal permeability but also enhances the expression of pro-inflammatory mediators. For example, Linsalata et al. found that IBS-D (IBS accompanied with depression) subjects exhibited an increased small-intestinal permeability, elevated levels of dysbiosis and pro-inflammatory cytokines, and abnormal changes in polyunsaturated fatty acid (PUFA) metabolism versus IBS-D subjects without depression [69]. In turn, disturbances in the GM composition may affect developing anxiety or mood disorders, leading to a vicious circle effecting and aggravating IBS symptoms. Johannes et al. showed that patients with IBS exhibited an increased psychological distress (65%), anxiety (31%), and depression (21%) that were associated with elevated abundances of *Proteobacteria* and *Bacteroidaceae* and decreased abundance of *Lachnospiraceae* [70].

Other studies revealed similarities in the GM composition of individuals with psychosomatic disorders and depression. For example, depressed patients often exhibit decreased abundance of *Faecalibacterium* and *Bifidobacterium* [71]. In a study conducted by Simren et al., individuals with IBS also exhibited a reduction in anti-inflammatory bacteria like *Lactobacillus* and *Bifidobacterium* and elevation in the levels of pro-inflammatory bacteria such as *Escherichia coli* and *Streptococcus* [72]. Yuan et al. reported that patients with ulcerative colitis and depression/anxiety exhibited reduced fecal microbial community richness and diversity, along with increased abundance of *Lactobacillales*, *Sellimonas*, *Streptococcus*, and *Enterococcus* and reduced abundance of *Prevotella_9* and *Lachnospira* [73]. Wu et al. found that patients with inflammatory bowel disease (IBD) and depression (IBDD) had reduced levels of *Bacteroides vulgatus (B. vulgatus*) versus patients with IBD without depression [74]. They also found that p-hydroxyphenylacetic acid (4-HPAA), a key metabolite of *B. vulgatus*, could reduce intestinal inflammation and improve depression-like behaviors in a mouse model of dextran sodium sulfate-induced colitis by increasing the expression of the tight junction protein claudin-5 in the vascular endothelium of the BBB [74]. While inflammation is a key outcome of GM dysbiosis in these diseases, GM dysbiosis is also linked to reduced generation of metabolites involved in epigenetic modifications. As another example, Nhu et al. found that depression in patients with fibromyalgia is associated with increased abundance of *Phascolarctobacterium* taxon and reduced abundance of anti-inflammatory and butyrate-producing bacteria such as *Faecalibacterium* taxon [75]. In another study, patients with fibromyalgia exhibited reduced bacterial diversity and decreased abundance of SCFA-producing bacteria, including the *Bifidobacterium* and *Eubacterium* genera involved in host neurotransmitter metabolism and modulation of immune function system [76]. Additionally, Kim et al. reported decreased bacterial diversity and reduced abundance of propionate-producing bacteria in fibromyalgia and thereby decreased concentrations of the stool propionate [77].

In this line, a systematic review, which includes 24 studies from 22 articles, indicated that patients with IBS exhibit increased levels of family *Enterobacteriaceae* (phylum *Proteobacteria*), family *Lactobacillaceae*, and genus *Bacteroides* and reduced levels of butyrate- and other SCFA-producing bacteria such as uncultured *Clostridiales* I, genus *Faecalibacterium* (including *Faecalibacterium prausnitzii*), and genus *Bifidobacterium* [78]. Other studies also provided experimental evidence that gut dysbiosis is associated with reduced production of SCFAs [79]. Furthermore, as reviewed elsewhere, several studies provided strong evidence that inflammation itself induces widespread epigenetic alterations [33]. Collectively, these studies indicate that gut-dysbiosis-induced decline in SCFA production and dysbiosis-induced inflammation may lead to epigenetic dysregulation identified in psychosomatic diseases, as addressed in the following sections.

## 3. Epigenetic Dysregulation of Genes Related to Gut Dysbiosis and Neuronal Functions in Patients with Psychosomatic Disorders

The main epigenetic mechanisms are classified into (i) DNA methylation, (ii) post-translational histone modifications, and (iii) non-coding RNAs, which contribute to the regulation of gene expression without altering the underlying DNA sequences [80,81]. DNA methylation is defined as the addition of a methyl group to cytosine in the context of cytosine–guanine dinucleotide (CpG) across the genome that mainly is linked to the suppression of gene expression [82]. Accumulating evidence has indicated that psychosomatic disorders are associated with alterations in DNA methylation of specific genes related to neuronal or brain function. For example, Mahurkar-Joshi et al. recently reported that the highest numbers of differentially methylated CpG sites in the blood cells of patients with IBS are relevant to genes involved in cell adhesion and neuronal pathways [83]. Another genome-wide methylation profiling reported 133 differentially methylated positions of genes linked to glutathione metabolism and oxidative stress (e.g., SSPO and GSTM5) in blood cells of patients with IBS [84]. Zhang et al., in another genome-wide methylation profiling of blood cells, showed that chronic negative stress gives rise to changes in DNA methylation of certain cardiac genes, which, in turn, accelerate pathologic remodeling of the heart. Their results showed altered methylation at specific genes linking to dilated cardiomyopathy (e.g., desmin, which codes a muscle-specific, type III intermediate filament protein) and adrenergic signaling of cardiomyocytes [85]. Gerra et al. reported a relationship between DNA methylation of *GRM2* (metabotropic glutamate receptor 2) gene and inflammation-related genes in women with fibromyalgia and depression [86]. In addition, DNA hypomethylation (exon 9) of brain-derived neurotrophic factor (BDNF) was found in blood cells of patients with chronic fatigue syndrome and comorbid fibromyalgia [87]. DNA hypomethylation of the SLC6A4 promoter was also reported in endoscopic gastric biopsies of patients with functional dyspepsia [88]. In a study by Goodin et al., they detected 6006 and 18,305 differentially methylated CpG sites (DMCs) among individuals with chronic low back pain, a psychosomatic disorder, and pain-free controls and found that most of the DMCs were hypomethylated and annotated to genes relevant to pain, including OPRM1, ADRB2, CACNA2D3, GNA12, LPL, NAXD, and ASPHD1 [89].

More studies addressing altered DNA methylation patterns related to brain function in patients with psychosomatic disorders are shown in Table 1. Generally, these methylation shifts propose altered stress-response gene regulation that may influence physiological reactivity to emotional signals. By activation or inactivation of potential pathways like serotonergic and pain pathways, the HPA axis, glutathione metabolism, oxidative stress, and inflammatory pathways, these epigenetic changes create a mechanistic bridge between psychological stressors and the somatic symptoms in psychosomatic disorders.

Histone modifications also contribute to regulation of gene expression, aggravating damage and causing long-term abnormal behaviors during psychosomatic disorders. In this process, condensation of the chromatin and a lower permissive state for transcription occur by removing acetyl groups by histone deacetylases (HDACs) from lysine residues on the tails of histone proteins [95]. Early-life stress increases the risk for the development of IBS that is associated with visceral pain and emotional comorbidities, like anxiety and depression. In a study conducted by Guan et al., amygdala-enhanced histone acetylation induced by stress in early life gave rise to visceral hypersensitivity and anxiety-like behaviors in rats [96]. In their study, rats exhibited elevated expressions of acetylated 9 residue of Histone 3 (H3K9) and protein kinase C zeta type (PKMζ) [96]. Qin et al. conducted a large-scale polygenetic risk scores-based analysis to identify the effect of GM × IBD interplays on the risk of depression and found several remarkable GM and IBD interactions and several candidate genes for depression phenotypes, such as HDAC7, GPM6A, VDR, and QRICH1. Interestingly, HDAC7 is a histone deacetylase involved in macrophage-mediated inflammatory response and elevating the release of pro-inflammatory mediators, such as IL-1β and Ccl2 [97]. Analysis of the histone methylation signature in intestinal epithelial cells from pediatric IBD patients has revealed enrichment of genes involved in immune regulation, metabolism, and cell survival pathways. In complementary mechanistic studies using germ-free mice colonized with commensal bacteria, it has been shown that the microbiota epigenetically regulates a substantial subset of these genes, many of which also exhibit altered expression in IBD patients [98]. Another study found that both microbiota composition and epigenetic profiles are altered in inflamed versus non-inflamed colonic regions in IBD patients [99].

Significant differences (*n* = 69 sites) in methylation patterns in women with fibromyalgia were also associated with genes involved in the regulation of histone acetylation, such as HDAC4 [100]. Taken together, the presence of histone modifications shows alterations in chromatin accessibility that may influence inflammatory responses, neural circuits involved in threat appraisal, pain perception, and mood regulation. These structural changes in the expression of certain genes may contribute to an elevated sensitivity to bodily sensations, a heightened inflammatory response, or altered autonomic tone and subsequently psychosomatic symptom profiles.

In addition to DNA methylation and histone modifications, miRNAs and other non-coding RNAs are known as post-transcriptional regulators of gene expression during psychosomatic disorders. For example, Matei et al. found differential expression in 45 miRNAs in IBD subjects, and 15 of these dysregulated miRNAs (let-7a-5p, let-7d-5p, let-7f-5p, let-7g-5p, miR-24-3p, miR-26a-5p, miR-26b-5p, miR-30b-5p, miR-107, miR-143-3p, miR-191-5p, miR-221-3p, miR-223-3p, miR-320b, and miR-339-5p) were linked to depression and/or anxiety in IBD [101]. In another study, Dobre et al. reported that miR-342-3p and miR-125a-5p may be considered possible biomarkers to discriminate between IBD subjects without depression and IBD with depression [102]. Hussein et al. found that subjects with fibromyalgia and persistent depressive disorder, insomnia, chronic fatigue syndrome, and primary headache disorder exhibited a higher serum level of micro-RNA-320a versus those without such symptoms [103].

Alterations in GM composition in psychosomatic disorders are associated with miRNA expression changes as well. For example, Flanagan et al. found that the expression of human-derived miRNA-21 was reduced in fecal samples from patients with IBS. Their results showed that miR-21 is capable of interplay with certain genera, including *Bacteroides*, *Limosilactobacillus*, *Ruminococcus*, or *Coprococcus*, and hence contributes to biosynthesis of indole and L-tryptophan, metabolites involved in regulation of inflammation and colonic motility [104]. It has also been shown that while the TNFA level is correlated with inflammation-related miRNA expression, such as miR-155 [105], miR-155-5p plays a key role in the pathogenesis of IBD by suppressing the expression of proteins involved in maintaining gut barrier integrity, including CLDN1 and ZO-1 expression, thereby contributing to gut dysbiosis [106]. Remarkably, as down-regulation of miR-16 expression was reported in the jejunum of patients with IBS [107], it is shown that miR-16 targets Toll-like receptor 4 (TLR4), inhibits TLR4/nuclear factor kappa B (NF-κB) signaling, and improves enterocyte viability and tight junction integrity while reducing apoptosis and cytokine production in an experimental animal model of IBS [108]. Olyaiee et al. also reported that reduced serum expression of mir-16 is linked to abnormal changes in bGM composition in subjects with IBS and Blastocystis infection compared to Blastocystis-negative IBS patients [109]. Furthermore, as up-regulation of miR-150 and miR-342-3p are involved in colonic motility, smooth muscle function, inflammatory pathways, and pain signaling in IBS [110], therapeutic effects of certain probiotics, including *L. fermentum* and *E. coli Nissle*, against colitis-associated dysbiosis and inflammation was shown to be mediated by restoration of the expression of miR-143 and miR-150 and reduced expression of MMP-2 and TNF-α [111].

Table 2 presents other human studies addressing altered miRNA expression generally related to gut dysfunction or inflammation in patients with psychosomatic disorders. In summary, dysregulated miRNAs, which function as post-transcriptional regulatory factors, influence key physiological processes, including serotonergic pathways involved in mood and behavior regulation, neuroinflammation, oxidative stress, neural plasticity, and stress hormone dynamics. These miRNA-mediated regulatory alterations may increase susceptibility to sustained physiological activation in response to psychological stress, creating a molecular context in which subjective distress manifests as persistent somatic symptoms.

**Figure 1:**
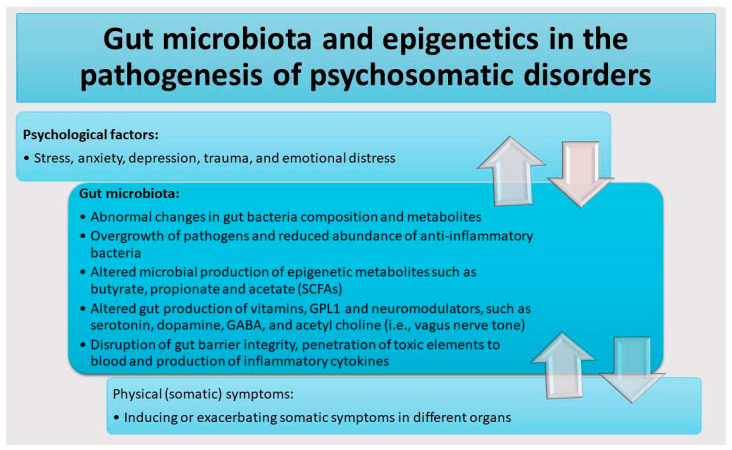
Association between psychological factors, gut dysbiosis, and physical (somatic) symptoms in psychosomatic disorders. Psychological factors like stress, anxiety, depression, trauma, and emotional distress may induce gut dysbiosis, cause abnormal gut microbiota (GM) composition, and alter the production of key epigenetic metabolites and neurotrasmiters. These GM-related abnormalities, in turn, can exacerbate physical (somatic) symptoms. Importantly, this correlation is bidirectional, as worsening somatic symptoms can further escalate psychological distress, driving the cycle observed in psychosomatic disorders.

An overview of the cascade of events through which psychological factors, such as stress, anxiety, depression, trauma, and emotional distress, may contribute to aggravating physical (somatic) symptoms by inducing abnormal changes in the composition of and epigenetic metabolites produced by gut bacteria is provided in Figure 1. Psychological factors like stress, anxiety, depression, trauma, and emotional distress can disrupt the GM profile. This disruption is reflected in an elevated abundance of pathogenic and pro-inflammatory bacteria, a decreased abundance of beneficial commensal species, and abnormal production of key epigenetic metabolites (e.g., butyrate and acetate involved in histone modifications or vitamins and choline essential for one-carbon metabolism), neurotransmitters (Table 1), and inflammatory factors (Table 2). These GM-related abnormalities, in turn, can exacerbate somatic symptoms across various organ systems, such as the brain, gut, skin, and cardiovascular system. Psychological factors may also trigger changes in GM composition, heightening systemic inflammation, or derangements in neuroendocrine pathways such as the HPA axis. These abnormalities are then observed as somatic symptoms influencing various body organs, such as the brain, gut, skin, and cardiovascular system. At the same time, the presence of persistent or distressing physical symptoms may influence psychological function. For example, chronic pain, fatigue, GI inflammation, or skin abnormalities often increase emotional distress and anxiety- or depressive-like behaviors.

## 4. Microbiota-Based Interventions Affecting Epigenome for Management or Treatment of Psychosomatic Disorders

Nutritional interventions that affect the GM may be considered one of the promising approaches in the treatment of psychosomatic disorders, especially IBS by mediation of epigenetic changes [118,119]. Given the involvement of gut microbiota dysbiosis and epigenetic aberrations in psychosomatic disorders, prebiotic interventions represent a key nutritional strategy capable of restoring both microbial imbalance and epigenetic dysregulation, while probiotics, postbiotics, and fecal microbiota transplantation offer additional alternative approaches for disease improvement.

**Prebiotics**

Prebiotics are food substrates that are selectively utilized by host microorganisms, enhancing the growth and activity of beneficial bacteria in the gut [120]. Non-digestible food components, such as certain fibers, oligosaccharides, and resistant starches, represent the main class of prebiotics [121]. These food components can be metabolized by the GM to generate bioactive metabolites, including SCFAs and other compounds, which exert diverse effects on host physiology, including epigenetic regulation [122]. For example, Lutz et al. unraveled how fiber-rich diets can improve mental health by affecting GM and epigenetic modifications [123]. Experimental studies have also shown that a traditional Chinese herbal formula has been found to alleviate intestinal and depressive symptoms in mice with IBS-D by regulating the intestinal IL-17-related signaling pathway ACT1/TRAF6/MAPKs/AP-1 and the DRD2/TH signaling pathway in the brain [124]. The protective effects of Xiaoyao San have been attributed to an increase in butyrate metabolite levels [125]. Dietary inulin can also improve constipation-induced depression- and anxiety-like behaviors by increasing the abundance of *Bacteroides* and *Proteobacteria*, reducing levels of *Muribacalum* and *Melaminabacteria*, increasing concentrations of SCFAs as epigenetic modifiers, suppressing inflammatory responses, and enhancing synaptic plasticity in the mouse brain [126]. Costunolide, a sesquiterpene lactone and a type of natural compound present in plants synthesized from acetate, could improve intestinal dysfunction and depressive symptoms in stress-induced IBS mice by suppressing low-grade colon inflammation, inhibiting the activation of mast cells, improving intestinal mucosal permeability, up-regulating 5-HT levels in hippocampal cells, inhibiting 5-HT metabolism, increasing the expression of colonic occludin, and reducing Claudin 2 expression [127]. In a study conducted by Baldi et al., treatment of patients with fibromyalgia syndrome with ancient Khorasan wheat was shown to alleviate the disease symptomatology by improving the inflammatory profile, elevating butyric acid levels, and increasing the abundance of *candidatus Saccharibacteria* and *Actinobacteria* while decreasing the abundance of *Enterococcaceae* [128].

A ketogenic diet (KD) is another type of nutritional intervention, during which a very low-carbohydrate, high-fat, and moderate-protein diet is capable of promoting ketone body production, such as beta-hydroxybutyrate, which not only acts as an epigenetic modulator but also serves as an alternative fuel, particularly for the brain [129,130]. In an animal model of colitis induced by dextran sulfate sodium (DSS), KD could alleviate IBD-like symptoms in mice by modulating GM composition (specifically increasing *Akkermansia* and reducing *Escherichia/Shigella*) and altering metabolite profiles. Remarkably, FMT from mice fed a KD could relieve DSS-induced colitis in recipient mice, suggesting a causal relationship [131]. KD has also been found to be a promising intervention for the management or treatment of psychosomatic disorders by regulation of the gut–brain axis. As an interesting example, in a study conducted by Orlando et al., Wistar rats were exposed to maternal deprivation as newborns and then fed with a standard diet (IBS-Std) or KD (IBS-KD) for ten weeks to examine its protective effects. Their results revealed that KD could mitigate the detrimental effects of stress in IBS animals by preventing the stress-induced increase in mucosal 5-HT without altering transporter or receptor levels, enhancing brain BDNF levels, and down-regulating TrkB [132]. In another study Chimienti et al., it was reported that treatment with KD for ten weeks could strongly hamper the detrimental impact of stress on gut mitochondrial biogenesis in a rat model of IBS by suppressing inflammation and oxidative stress via the activation of the PPAR-γ/PGC-1α axis [133].

**Probiotics**

The beneficial effects of probiotics in subjects with IBS are associated with increasing concentrations of SCFAs such as acetate, propionate, and butyrate as epigenetic modifiers, repairing the intestinal barrier, suppressing neuroinflammation, decreasing pain and symptom severity scores, and improving mental health quality [134,135,136]. Among probiotics, bacteria capable of producing epigenetic metabolites, particularly *Bifidobacteria* and *Lactobacillus* species, have been reported to be helpful in the treatment of psychosomatic disorders [137]. The beneficial effects of probiotics also are connected to modulating the expression of miRNAs such as miR-144 that are involved in regulation of intestinal epithelial permeability in IBS-D rats [111,138,139]. As an interesting example, Qiuke Hou et al. found that *Lactobacillus* casei LC01 enhances intestinal epithelial barrier integrity by decreasing miR-144 levels and subsequently overexpression of the tight junction proteins occludin and ZO-1 [140].

Staudacher et al. found that co-administration of a multistrain probiotic and dietary restriction of fermentable carbohydrates (a low-FODMAP diet) in IBS subjects alleviated symptoms and elevated *Bifidobacterium* species involved in production of epigenetic modifiers versus a placebo [141]. Groeger et al. reported that the stress response is a core driver of IBS symptoms, and treatment of IBS patients with a probiotic named “COMBO” product (*Bifidobacterium longum* 1714^®^ and *Bifidobacterium longum* 35624^®^, two supporters of butyrate production in the gut) for 8 weeks improved IBS symptoms and decreased HADS-D, HADS-A score, and circulating levels of TNF-α [142].

Ustaoğlu et al. investigated the effects of the *L. rhamnosus* (LGG) as a probiotic supplement in addition to a low-fermented-oligosaccharide, -disaccharide, -monosaccharide, and -polyols (low-FODMAP) diet on symptoms and quality of life in IBS subjects and found that it could alleviate IBS Symptom Severity Score (IBS-SSS) and anxiety and depression scores [143]. Martina et al. reported that the probiotic *Bifidobacterium longum* (BL) NCC3001 could alleviate depression and anxiety and reduce brain emotional reactivity in IBS patients by elevating the concentrations of butyric acid, tryptophan, N-acetyl tryptophan, glycine-conjugated bile acids, and free fatty acids. Interestingly, they also found that lower anxiety and depression scores in treated patients are linked to butyric acid concentration as an epigenetic metabolite [144]. Mullish et al. found that a Lab4 probiotic, which consisted of four bacterial strains, could reduce IBS-SSS and anxiety and depression scores in IBS patients by reshaping the GM composition and increasing levels of butyrate-producing bacteria [145]. Sarkawi et al. reported that two bottles of cultured milk drink containing one billion cfu (Colony-Forming Units) of dual-strain *lactobacillus* for 12 weeks could improve depressive-like behaviors in IBS subjects by increasing serotonin serum levels [146]. In a study by Çİn et al., a probiotic containing SCFA-producing bacteria, including *Lactobacilli (Lactobacillus acidophilus L1*, *Lactobacillus rhamnosus liobif*, *Bifidobacterium longum*, and *Saccharomyces boulardii*), could reduce Beck Depression Index (BDI), Beck Anxiety Index (BAI), and Pittsburgh Sleep Quality Index (PSQI) scores in subjects with fibromyalgia syndrome [147]. In a study conducted by Dao et al. using multispecies psychobiotics, a class of probiotics, prebiotics, or other microbiota-targeted interventions capable of exerting beneficial effects on mental health by affecting the gut–brain axis, for 2 months could alleviate anxiety and depression symptoms in subjects with chronic GI symptoms [148]. In a recent study, Wang et al. examined the efficiency of the psychobiotic *Bifidobacterium breve* BB05 in managing psychosomatic diarrhea in college students by modulating GM and found that BB05 supplementation could improve the symptoms of diarrhea, anxiety, and depression by restoring GM composition, particularly elevating levels of butyrate-producing bacteria like *Bifidobacterium* and *Roseburia* [149].

**Postbiotics**

Postbiotics are functional bioactive metabolites and cellular components derived from microbial fermentation, such SCFAs, peptides, and enzymes, that can modulate host physiology and health-promoting immune responses [150,151,152]. Abnormal changes in SCFAs have been connected to both depressive symptoms and gut symptoms in young adults [153]. For example, there is a negative association between butyrate and propionate levels and depressive symptoms [153]. Srivastava et al., examined the efficacy of the probiotic *Bifidobacterium longum* (ES1) and the postbiotic heat-treated *Bifidobacterium longum* ES1 (HT-ES1) in alleviating GI symptoms of subjects with IBS-D and found remarkable reductions in IBS-SSS scores and anxiety symptoms [154]. In a study published by Firoozi et al., sodium butyrate supplementation in patients with active ulcerative colitis could improve psychological symptoms by reducing HADS anxiety and HADS depression scores [155]. Moreover, protective effects of sodium butyrate in patients with active ulcerative colitis can be associated with up-regulation of circadian-clock genes, improving sleep quality, and suppressing inflammation [156].

In a mice model of ulcerative colitis, sodium butyrate was capable of suppressing intestinal oxidative damage and neuroinflammation in the prefrontal cortex by increasing the expression of tight junction proteins and restoring intestinal barrier integrity [157]. Lacidophilin tablets (LH), a postbiotic composed of defatted milk by *Lactobacillus acidophilus*, which includes L-lactic acid, D-lactic acid, and seven SCFAs (acetic acid, propionic acid, butyric acid, isobutyric acid, valeric acid, isovaleric acid, and caproic acid), could also alleviate symptoms in a mouse model of low-grade colitis [158]. In a recent study by Fan et al., a 2-week treatment with LH in rats with IBS reshaped the structure and composition of the gut microbiota, reduced visceral sensitivity and intestinal motility, alleviated anxiety- and depression-like behaviors, up-regulated the expression of *Muc2*, *Claudin1*, and *Occludin*, and down-regulated the expression of inflammation-related genes [159]. Elmaaboud et al. have also shown that the antidepressant effect of sodium butyrate in a mouse model of reserpine-induced fibromyalgia is related to increased concentrations of spinal cord dopamine and serotonin, elevated levels of anti-inflammatory cytokines IL-4 and TGF-β1, and synaptophysin expression but also to a decrease in hippocampal NF-κB levels [160].

**Fecal microbiota transplantation (FMT)**

FMT is an approach in which stool from a healthy donor is transferred into a patient’s GI tract for restoring the balance of bacteria [45,161]. FMT also can be transferred from a patient into healthy animals to induce psychosomatic disorders and abnormal changes and behaviors for experimental research to support cause–effect relationships [62,162]. FMT may be considered a promising strategy for ameliorating anxiety and depression behaviors and the IBS-SSS score in IBS-D (diarrhea-predominant irritable bowel syndrome) subjects [163,164]. In this field, Lin et al. found that the protective effects of FMT in human IBS-D subjects are connected to a decrease in the abundance of *Faecalibacterium*, *Eubacterium*, and *Escherichia* and the concentration of isovaleric acid and valeric acid, short- to medium-chain fatty acids, as epigenetic modifiers [165]. A 6-months treatment with FMT in patients with fibromyalgia also resulted in lower symptom severity and Hospital Anxiety and Depression Scale scores, which were associated with elevated amounts of serotonin and gamma-aminobutyric acid glutamate but a reduced level of glutamate [166].

**Figure 2:**
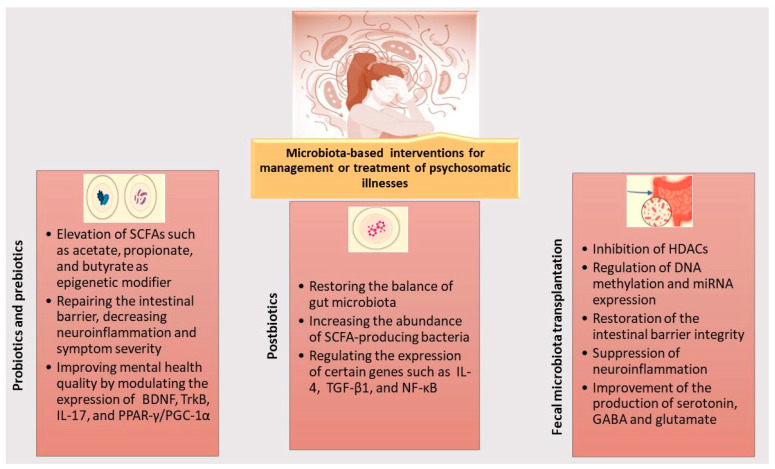
Schematic illustration of microbiota-based interventions influencing epigenome and subsequently gene expression for management or treatment of psychosomatic disorders. These microbiota-based interventions increase concentrations of SCFAs (e.g., acetate, propionate, butyrate), improve gut barrier integrity, suppress systemic and neuroinflammation, and normalize the gut microbiota by promoting SCFA-producing species. They also have demonstrated ability for alleviating both psychological and somatic symptoms by enhancing the production of neurotransmitters and regulating the expression of important neurobiological and immunoregulatory markers (BDNF, TrkB, IL-17, PPAR-γ/PGC-1α, IL-4, TGF-β1, and NF-κB) through inhibiting HDACs and modulating DNA methylation and miRNA expression.

Figure 2 shows how different types of microbiota-based interventions are capable of improving psychosomatic disorders by replenishing abnormal GM composition, enhancing concentrations of beneficial epigenetic metabolites, and controlling gene expression. For example, probiotics, prebiotics, and postbiotics are capable of elevating the production of SCFAs such as acetate, propionate, and butyrate, as well as choline and key vitamins, which are important or essential for epigenetic modifications. These interventions also contribute to reinforcing gut barrier integrity, thus inhibiting systemic and neuroinflammation and alleviating symptom severity. FMT is also capable of exerting broad regulatory effects across microbial, epigenetic, and neuroimmune pathways by enhancing the production of key metabolites and neurotransmitters, restoring intestinal barrier integrity, and subsequently reducing permeability and systemic inflammatory responses.

**Electrophysical therapies to temper gut dysbiosis and inflammation and improve brain function**

Electroacupuncture is a modern method that combines traditional acupuncture with electrical stimulation by passing a small electric current through acupuncture needles placed at specific points on the body [167,168]. The electrical current stimulates nerve fibers, promotes endorphin release, and enhances blood circulation [169]. While experimental studies have provided evidence that, similar to physical exercise, this method affects DNA methylation and gene expression of many genes in skeletal muscles [170], it also affects the GM structure. For example, a study by Zhou et al. supports the idea that IBD-related anxiety and depression in rats can be alleviated by electroacupuncture through elevating the abundance of butyrate- and acetate-producing bacteria, including *Ruminococcaceae*, *Phascolarctobacterium*, and *Akkermansiaceae*, associated with overexpression of l-glutamine and gamma-aminobutyric acid (GABA) in the hippocampus region, reducing IL- 1β levels, and inhibiting the TLR4/ NF-κB signaling pathways and NOD-like receptor protein 3 (NLRP3) inflammasomes [171]. In a recent human study, a one-month electroacupuncture therapy regime could also improve clinical symptoms and gut dysbiosis and normalize neurotransmitter-related metabolic pathways in patients with IBS and constipation [172]. The beneficial effects of electroacupuncture are also linked to regulating the expression of miRNAs. For example, Hou et al. provided preliminary evidence that electroacupuncture could enhance intestinal barrier integrity by inhibiting mast-cell-derived exosomal miR-149-5p and miR-22-5p, indicating these miRNAs as core mediators of barrier dysfunction in IBS-D [173].

Percutaneous electrical nerve field stimulation (PENFS) is another promising therapeutic approach to alleviate symptoms of IBS through plausible vagal neuromodulation and influencing GM composition via brain–GM signaling. As an interesting example, Bora et al. found that 4 weeks of PENFS therapy could ameliorate IBS severity, visceral sensitivity, and functional disability scores by enhancing α-diversity and increasing abundance of *Blautia*, a butyrate-producing bacterium, in excellent responders [174]. Castillo et al. found that PENFS therapy could alleviate pain and psychological outcomes in IBS patients by decreasing *Clostridial* species and long-chain fatty acid-dependent microbial activity [175].

Physical exercise is also capable of affecting gut motility, permeability, immune function, and GM composition depending on its intensity and duration [176,177]. Regular moderate exercise may improve the quality of life in IBS patients. Additionally, Chao et al. found that combination therapy using online yoga, mindfulness, and probiotics could improve disease severity in IBS patients by enhancing physical fitness, mental health, and favorable alterations in GM composition, including reduction in *Klebsiella* bacterial strains [178].

## 5. Conclusions and Perspectives

Psychosomatic disorders are conditions in which mental states are capable of influencing physical well-being and vice versa and manifest as physical and mental symptoms. The GM revolution has opened new avenues to investigate the relationship between the brain and the GM in the context of understanding and managing/treating psychosomatic disorders via epigenetic mechanisms. This review provides a detailed overview of findings regarding changes in the GM and their epigenetic metabolites in subjects with psychosomatic disorders. These findings show that the pathophysiology of psychosomatic disorders is associated with epigenetic aberrations and alterations in the GM. Findings from various articles showed anomalous changes in epigenetic markers, diversity metrics, microbial relative abundance, and epigenetic metabolites in patients and animal models. The most consistent findings across studies were increased levels of pro- inflammatory bacteria like *Streptococcus* and *Eggerthella* and reduced levels of anti-inflammatory butyrate-producing bacteria in psychosomatic disorders. Such changes in the above-mentioned bacterial genera and their epigenetic metabolites were found to be linked to higher severity of physical and mental symptoms. Although microbiota-based interventions show promising outcomes, the findings of probiotic trials have been somewhat discrepant. Today, the results of prebiotics and FMT appear to be too limited to depict definitive and precise conclusions. Furthermore, even with rigorous donor screening, there remains a risk of transmitting harmful bacteria, viruses, fungi, or parasites that go undetected during testing to the recipients. Therefore, more future studies should be implemented to increase our knowledge on useful as well as harmful epigenetic alterations and bacterial species in larger populations. Benefits include the modulation of immune responses, enhancement of intestinal barrier integrity, regulation of neurotransmitter synthesis, and improvement of mitochondrial and metabolic functions by SCFAs, tryptophan metabolites, and bile acid derivatives. Conversely, detrimental impacts may stem from derangements in microbial metabolism, which, in turn, gives rise to overgrowth of pathogens and the accumulation of toxic metabolites (e.g., lipopolysaccharides, ammonia, or secondary bile acids) that initiate inflammation, oxidative stress, or epigenetic aberrations connected to psychosomatic disorders. Although this review highlights the complex bidirectional relationships among gut dysbiosis, epigenetic alterations, and psychosomatic disorders, a solid cause–effect relationship among these factors has yet to be firmly established. Much of the available evidence is derived from cross-sectional or animal studies, which limit the ability to determine temporal sequences of events or to discern whether microbial and molecular changes act as initiating drivers or arise secondarily to the disorder. Moreover, psychosomatic conditions are influenced by a wide range of contributing factors, including dietary habits, stress, medication use, and illness-related lifestyle changes that independently modulate both the GM and epigenetic signatures. While transplantation of disease-related microbiota from humans or model animals into healthy animals could induce disease-like phenotypes, providing supportive evidence for the microbiota contribution to psychosomatic disorders [127], more longitudinal, mechanistic, and intervention-based studies are still needed to more accurately delineate causal pathways.

The absence of standardized protocols and the limited availability of robust human studies, particularly double-blind, randomized controlled trials, currently restrict the ability to propose evidence-based therapeutic recommendations. Therefore, future research incorporating well-designed mechanistic studies and double-blind, randomized controlled trials is crucial to validate these proposed interplays and therapeutic pathways. Moreover, many trials are conducted with small sample sizes and without a priori power calculations for clinically relevant outcomes, or they rely on a heterogeneous set of outcome measures (e.g., clinical scores, biomarker changes, self-reported symptoms) that vary across studies. In existing clinical investigations, limited sample sizes reduce the ability to detect true associations and increase the likelihood of unreliable effect estimates. Additionally, non-uniform or self-reported endpoints (such as self-reported mood or gastrointestinal symptoms) introduce substantial noise and further compromise reproducibility.

It is also important to note that most current findings related to microbiota-based interventions and microbial metabolites have been derived from studies in small animals and rodents, which differ considerably from human physiological conditions. Thus, future studies should focus on beneficial and detrimental effects of GM-derived metabolites and specific nutrients in patients with psychosomatic disorders. However, before conducting direct human studies, investigations on human iPSC-derived organoid models may be considered promising platforms to clarify their beneficial or harmful effects under controlled conditions that more closely mimic human physiology. In fact, advanced three-dimensional organoid systems derived from human intestinal, hepatic, brain, and pancreatic tissues provide a versatile platform to investigate the complex interplay among the GM, epigenetic regulation, endocrine signaling, and immune–neural communication underlying psychosomatic disorders. Intestinal organoids enable direct assessment of microbial–epithelial interactions, barrier function, and microbial extract or active metabolite (e.g., SCFAs) effects on epigenetic and immune signaling pathways [179]. For instance, Farhadipour et al. showed that in human intestinal organoids, microbial-derived SCFAs can alter stem cell fate by inhibiting HDACs, leading to increased differentiation toward absorptive enterocytes and reduced intestinal permeability [180]. In another noteworthy study, co-culture of cerebral organoids with non-pathogenic and pathogenic bacteria revealed that the pathogenic strains disrupt energy metabolism and compromise the structural integrity of the organoids [181]. Liver organoids allow for modeling of metabolic, detoxification, and inflammatory responses to microbial-derived or stress-induced metabolites, linking gut dysbiosis to systemic organ dysfunction [182]. Pancreatic organoids provide a valuable platform to investigate the gut–pancreas axis and its influence on neuroendocrine and immune–metabolic regulation, an interaction increasingly recognized in psychosomatic and metabolic disorders [183,184]. The integration of multi-organoid systems, potentially via microfluidic or organ-on-chip platforms, may create a next-generation framework to dissect mechanistic cascades from microbial perturbations to epigenetic modulation, organ-specific dysfunction, and downstream psychosomatic phenotypes. Employing these organoid-based approaches in future research may facilitate mechanistic insight, biomarker discovery, and development of microbiota- and epigenetic-targeted therapeutic strategies, bridging in vitro modeling and clinical applications.

## Figures and Tables

**Table 1:** Altered DNA methylation in patients with psychosomatic disorders.

Psychosomatic Disorders	Candidate Gene(s)/Targeted Pathway	Sample/Number of Participations	Key Findings	Ref.
Multisomatoform disorder (MSD)	TRPA1/pain pathway (mechanical pain sensitivities)	Blood/151 patients and 149 matched healthy controls	Higher methylation is linked to higher pain thresholds; childhood trauma affects TRPA1 promoter methylation	[90]
Somatoform disorder (somatization)	SLC6A4 gene/serotonergic pathways involved in mood and behavior regulation	Peripheral blood/148 monozygotic twin subjects	DNA methylation SLC6A4 of correlates with somatization symptoms; higher methylation in women vs. men	[91]
Functional dyspepsia	SLC6A4 gene/serotonergic pathways	Endoscopic gastric biopsies/79 patients vs. 78 controls	Lower SLC6A4 promoter methylation in patients (*p* = 0.04)	[88]
MSD	Leptin/hypothalamic–pituitary–adrenal (HPA) axis and pain pathway	Blood/151 patients and 149 matched healthy controls	Hypomethylation in female patients (CpG C-289) vs. controls (*p* < 0.05)	[92]
Somatic symptom disorder (SSD)	NR3C1/HPA axis	Saliva/34 children with SSD and 29 age- and sex-matched controls	Age-related differences in NR3C1 methylation (*p* < 0.05); exon 1F higher methylation in children aged 13 or older; methylation correlates with psychological symptoms in children under 13	[93]
Functional movement/conversion disorder (FMD)	Genes of antigen presentation pathway and GABA receptor signaling/pathways implicated in chronic stress and pain	Peripheral blood/57 patients with FMD and 47 healthy controls	Functional motor symptoms are linked to genome-wide DNA methylation variation; association between childhood abuse in females and distinct epigenetic signatures	[94]
Irritable bowel syndrome (IBS)	Certain genes such as SSPO and GSTM5/glutathione metabolism and oxidative stress	Blood cells (PBMCs)/27 IBS and 23 age- and sex-matched controls	133 differentially methylated positions of genes linked to glutathione metabolism and oxidative stress (e.g., SSPO and GSTM5) (*p* < 0.05)	[84]
Chronic fatigue syndrome and comorbid fibromyalgia	BDNF/BDNF signaling pathway	Blood/28 patients and 26 matched controls	Lower BDNF DNA methylation in exon 9 (*p* = 0.009)	[87]

**Table 2:** Altered miRNAs in patients with psychosomatic disorders.

Psychosomatic Disorders	miRNAs Analyzed	Samples/Targeted Pathway	Main Finding in Patients	Ref.
Irritable bowel syndrome (IBS)	miR-150 and miR-342-3p/AKT2	Whole-blood samples/inflammatory and pain pathways	Up-regulation of miR-150 and miR-342-3p in IBS is involved in inflammatory pathways, colonic motility, smooth muscle function, and pain signaling (*p* < 0.05)	[110]
IBS	miR-16 and miR-103	Jejunum/serotonergic pathways involved in mood and behavior regulation	Down-regulation of miR-16 and miR-103 and hence dysregulation of HTR4 gene (*p* < 0.05)	[107]
IBS	miRNA-219a-5p and miRNA-338-3p	Sigmoid colon/mitogen-activated protein kinase (MAPK) signaling pathway involved in immune response	Down-regulation of miRNA-219a-5p dysregulates proteasome/barrier function genes and increases intestinal epithelial cells permeability; down-regulation of miRNA-338-3p affects expression of MAPK-signaling genes (*p* = 0.026 and *p* = 0.004)	[112]
IBS	miR-16	Serum/inflammatory pathway	miR-16 targets TLR4 and inhibits TLR4/NF-κB signaling and lncRNA XIST, improving enterocyte viability and tight junction integrity, reducing apoptosis and cytokine production (*p* < 0.05)	[108]
IBS	Analysis of eight miRNAs following TaqMan low-density array screening	Serum/multiple pathways, such as TGF-β, p53, insulin, B-cell receptors, GnRH, and adipocytokine	Up-regulation of several miRNAs (miR-1305, miR-575, miR-149-5p, miR-190a-5p, miR-135a-5p, and miR-148a-3p) and down-regulation of some others (miR-194-5p, miR-127-5p) are linked to IBS pathogenesis (*p* < 0.05)	[113]
IBS	miR-155-5p	Human (and mouse) colon samples/pathways linked to intestinal inflammation and epithelial barrier	Up-regulation of miR-155-5p (*p* < 0.01) and reduced levels of tight junction proteins, including CLDN1 and ZO-1	[106]
IBS	miR-148	Fasting venous blood	Association between miR-148 expression and the severity of IBS (*p* < 0.05)	[114]
Conversion disorder/functional neurological disorder	miR-146a, miR-155, miR-21 and miR-132	Blood/inflammatory pathway	TNFA level is linked to inflammation-related miRNA expression (miR-146a and miR-155); miR-21 and miR-132 levels are linked to vascular inflammation	[105]
Psychological cardiovascular diseases	hsa-miR-1976 and hsa-miR-4685-3p	Fasting venous blood/neurotrophins pathways	Reduced miRNA expression targeting PI3K-Akt and neurotrophins pathways linked to cardiovascular and mental health (*p* < 0.05)	[115]
Fibromyalgia syndrome	Eleven miRNAs	Plasma	SF-36 mental symptoms directly correlate miR-142-3p and inversely with miR-320a and b (*p* < 0.001 and *p* < 0.01)	[116]
Fibromyalgia syndrome	MitomiR-145-5p	PBMCs/oxidative stress	Elevated mitomiR-145-5p in fibromyalgia and depression vs. controls (*p* = 0.0010)	[117]

## Data Availability

No applicable.
